# RDF2Graph a tool to recover, understand and validate the ontology of an RDF resource

**DOI:** 10.1186/s13326-015-0038-9

**Published:** 2015-10-23

**Authors:** Jesse CJ van Dam, Jasper J Koehorst, Peter J Schaap, Vitor AP Martins dos Santos, Maria Suarez-Diez

**Affiliations:** Laboratory of Systems and Synthetic Biology, Wageningen University, Dreijenplein 10, Wageningen, 6703 HB The Netherlands; LifeGlimmer, GmbH, Markelstrasse 38, Berlin, Germany

**Keywords:** Data integration, RDF, Databases, Semantic web, Visualization, SPARQL, OWL, Ontology

## Abstract

**Background:**

Semantic web technologies have a tremendous potential for the integration of heterogeneous data sets. Therefore, an increasing number of widely used biological resources are becoming available in the RDF data model. There are however, no tools available that provide structural overviews of these resources. Such structural overviews are essential to efficiently query these resources and to assess their structural integrity and design, thereby strengthening their use and potential.

**Results:**

Here we present RDF2Graph, a tool that automatically recovers the structure of an RDF resource. The generated overview allows to create complex queries on these resources and to structurally validate newly created resources.

**Conclusion:**

RDF2Graph facilitates the creation of complex queries thereby enabling access to knowledge stored across multiple RDF resources. RDF2Graph facilitates creation of high quality resources and resource descriptions, which in turn increases usability of the semantic web technologies.

**Electronic supplementary material:**

The online version of this article (doi:10.1186/s13326-015-0038-9) contains supplementary material, which is available to authorized users.

## Background

In the life sciences, high-throughput technologies deliver ever-growing amounts of heterogeneous (meta) data at different scales, which are produced, stored and analysed in both structured and semi-structured formats. Systems Biology is an integrative discipline that uses various integration strategies to model and discover properties of biological systems. Integration and analysis of heterogeneous biological data and knowledge require efficient information retrieval and management systems and Semantic Web technologies are designed to meet this challenge [1].

The RDF data model is a mature W3C standard [2, 3] designed for the integrated representation of heterogeneous information from disparate sources and it is proving effective for creating and sharing biological data. RDF is not a data format, but a data model for describing resources in the form of self-descriptive subject, predicate and object triples that can be linked in an RDF-graph. Integration of heterogeneous data from different sources in a single graph relies on using retrievable controlled vocabularies, which is essential to access and analyse integrated data [4]. Once data sources are converted into the semantic Web, SPARQL [5, 6] can be used to query multiple of these resources, simultaneously or consecutively, without further modifying any of them.

Widely used biological resources such as Reactome, ChEBI and UniProt, among others, [7–10] have been transformed into the RDF data model and the Bio2RDF [11] project has transformed a large set of additional sources, such as the NCBI GenBank files [12], DrugBank [13] and InterPro database [14]. Additionally, there are on-going efforts to develop tools providing results in this data model, such as the Semantic Annotation Platform for Prokaryotes, SAPP, (J. Koehorst, J. van Dam et al. personal communication) that provides genome functional annotation in the RDF data model.

These RDF resources can be readily queried with SPARQL. Constructing SPARQL queries requires that the user has a mental representation of the underlying structure of the resource. The structure of a resource is the set of object types and their relationships, i.e. the explicit representation of the predicates linking different classes. This structure represents the set of semantic constraints embedded in the resource. In a biological database containing information on biochemical reactions, genes and their identifiers are linked to proteins; proteins are linked to EC numbers; EC numbers are connected to reactions that involve metabolites as products and substrates. To retrieve information pertaining metabolites and genes, the SPARQL query has to obey the specific network topology linking these types of objects. RDF data sources do not need a predefined scheme so that new data types can be added at any time expanding the underlying structure. If the modifications in the underlying structure generated by this new data are not known, linked information cannot be retrieved. Not having a clear idea of the underlying structure makes querying an RDF resource inefficient, time consuming, or even impossible.

The structure of a resource can be either retrieved through manual queries or it can be provided by the data publishers in the documentation of the resource. This structural information can be encoded using Web Ontology Language (OWL) files [15]. OWL was created as a description logic language and it is intended for automatic reasoning; nevertheless, its axioms can also be used to construct a graphical overview of the described resource [16]. However, the OWL standard does not require all axioms necessary for such reconstruction. Examples of necessary axioms not obligated by this standard are *object all values from* and *data all values from*. In some of the resources created by the Bio2RDF project these axioms (*object all values from* and *data all values from*) are not provided. Furthermore, the ontology generation process is, at best, semi-automatic, time consuming and error-prone. Errors might also accumulate due to the conversion code used to generate the RDF resource, as the triple generating code can contain lexical errors in predicate definition such as typos, inconsistent usage of upper and lower case, or misspelled words, thereby populating a resource containing information on proteins with information on “porteins”, which describes proteins associated to transmembrane transport. These errors lead to descriptions of the intended content of the resource rather than of its actual content.

Shape Expressions (ShEx) is a standard to describe, validate and transform RDF data. One of the goals of this standard is to create an easy to read language for the validation of instance data, however, it is still being developed and no final recommendation is yet available [17–19].

Computational tools able to reconstruct the structure of RDF resources are thus required to i) facilitate query writing and to ii) enable data providers to verify the structural integrity of their resource. To our knowledge, no such tool, able to automatically recover the structure of the resource and the associated multiplicity of the predicates, exist. Semscape [20] is an already existing Cytoscape [21] app that is able to retrieve to some extent the structure of the resource. However, it has limited recovery and simplification capabilities, leading to unreadable hairballs for larger structures. Furthermore, additional statistical information about the classes and links is not retained. Here we present **RDF2Graph**, a tool to automatically recover the structure of an RDF resource and to generate a visualization, ShEx file and/or an OWL ontology thereof. These can be used to write SPARQL queries or to verify (generated) RDF resources.

## Implementation

**Fig. 1:**
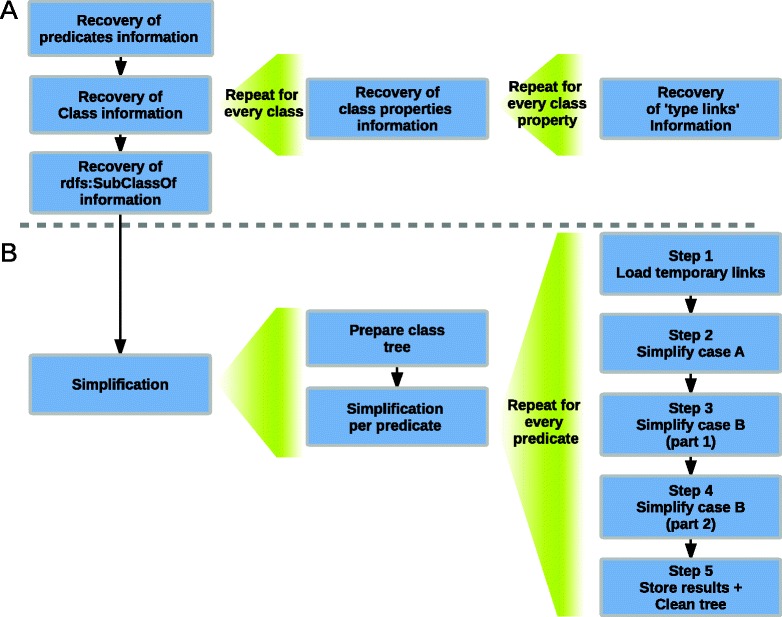
Overview of the structure recovery process. **a** Recovery of the information on predicates, classes, class properties, *unique type links* and class hierarchy **b** Simplification of the structure leading to a neat visualization by preventing the formation of an unreadable hairball

RDF2Graph performs two distinct processes to retrieve the structure of a resource. Initially, there is a recovery of all classes, predicates and *unique type links* together with their associated statistics. In the second stage there is a simplification step to arrive to a neat structural overview. A simplified overview of the complete process is given in Fig. 1.

A *type link* is defined as a link joining a subject class type to an object class or value data type, via a predicate. A *unique type link* is defined as a unique tuple: *type of subject, predicate, (data)type of object*. For the triple <:BRCA1, :locatedOn, :chromosome17> the *type link* is <:Gene, :locatedOn, :Chromosome>. When considering the full resource, all *type links* <:Gene, :locatedOn, :Chromosome> correspond to the same *unique type link*. In the triple <:Adam :hasSon :Bob> the *type link* is <:Person, :hasSon, :Person>.

The multiplicity of a *unique type link* describes the number of instances connected to each other. The forward multiplicity can be: i) *One-to-one* (also denoted: 1..1) each source instance has exactly one reference to the target; ii) *One-or-many* (1..N) each source instance has one or more references to the target; iii) *Zero-or-one* (0..1) some source instances have at most one reference to the target; iv) *Zero-or-many* (0..N) some source instances have one or even more than one reference to the target. Similarly, for the reverse multiplicity the roles of target and source are inverted. In the previous examples, the forward multiplicity of the *unique type link* <:Gene, :locatedOn, :Chromosome> is (1:1) since each human gene is associated to one and only one chromosome, whereas the reverse multiplicity is (1..N) since a chromosome contains many genes. In the second case <:Person, :hasSon, :Person>, the forward multiplicity is (0..N) since there is no limitation to the number of sons a person may have; in this case the reverse multiplicity is (*N* = 2..1) given that each son has two parents.

The initial recovery process is performed through a series of SPARQL queries on the selected endpoint. Detailed information about the SPARQL queries and the queries themselves are provided in **RDF2Graph**’s documentation. These queries can be adapted to change the introduced limitations and to customise the tool for specific end points. The queries can be limited to reduce the running time since this process can take between a few minutes for a resource with a million triples, to several days for a resource with 16 billion triples, such as the RDF version of UniProt, as described in the ‘Results’ section. However the limitation in the number of retrieved triples may lead to incompleteness of the recovered structure, since some *type links* could be missed. This may cause that for some *unique type links* not all *type links* are retrieved, which can cause errors in the calculation of the multiplicities (forward and/or reverse). It may also lead to some *unique type links* not being identified if no *type links* associated to them are found. Therefore, we advise caution when using these limitations.

**Fig. 2:**
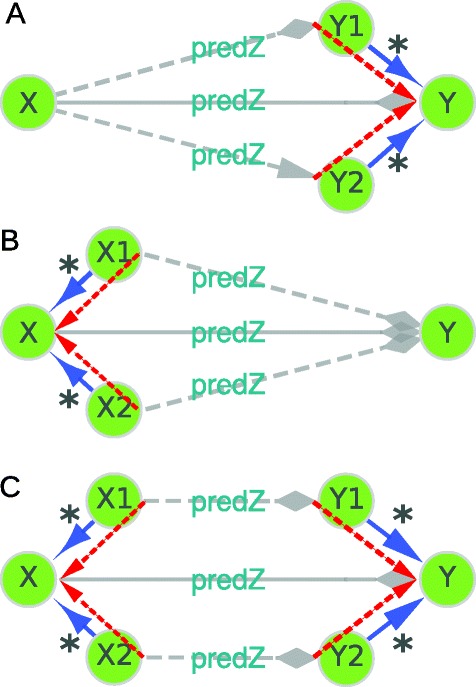
Graph simplification by merging of links and classes. Overview of the possible cases considered in the simplification step. *: Classes X1 and X2 are either equal to X or (indirect) subClassOf X, same applies to Y1, Y2 and Y. **a** Merging of two links in Class X that link to 2 subclasses of Class Y. **b** Merging of 2 links in 2 subclasses of class X. **c** Merging of two links in 2 subclasses of class X that link to 2 subclasses of Class Y

After the initial recovery of *type links* and *unique type links*, a simplification process follows, in which *type links* with a common parent class for either the subject or object types are merged. These process proceeds in a pairwise manner, so that at each iteration only two *unique type links* sharing either the subject type or the object (data)type are considered. If more than two *unique type links* are present, the first two are merged, and their result is combined with the next one and so on until all have been considered. Therefore, only two *unique type links* at a time are merged. Figure 2 represents the cases that need to be considered when analysing two *unique type links*. In principle, other cases involving the “sameAs” relationship could appear, but in our approach, the “subClassOf” relationship also includes the “sameAs” relationship, which reduces all possible cases to the ones represented in Fig. 2.

This process also allows the identification of *concept classes*. A *concept class* is defined as a class that has no instances and no subclasses with some instances. A typical set of examples of concept classes are all the GO classes in the GO database [22]. This concept is needed to support the exclusion of them in the network view as they have little value for the structural overview and will overcrowd the network visualization.

All classes identified in the recovery process and associated *subclassOf* links are loaded into a memory based directed graph. This class tree is then used in the merging process. During the merging process five steps are executed per retrieved predicate. Step 1 is the initialization; step 2 performs the merging in case A and steps 3 and 4 are used for case B, whereas case C is the combination of cases A and B from Fig. 2; step 5 is the fictionalization step.

The following definitions of *shared* types and *child of* classes are used. Two types are *shared* if i) both are the same, ii) one is a parent class of the other, or iii) both have a common parent class in the class tree. A *child of class* is defined as follows: Class K is a *a child of* class L if either class K is equal to class L or class K is a (non)direct subclass of class L.

- Step 1: For each *unique type link* found for the predicate currently processed a temporary link is added to the *type of subject*, which links to the *(data) type of object*. In this way a temporary link between both types is defined.
- Step 2: For each class in the class tree all temporary links are copied to the respective parent class(es). Then, occurrences of case A from Fig. 2 are simplified by performing a search for pairs of temporary links which both point to a *shared* type. If found, the temporary links are merged and replaced by a new temporary link pointing to the common parent class.
- Step 3: This step is executed as a per class recursion breadth first process over the class tree. For each temporary link of the currently processed class the number of direct ’child’ classes is counted if they have at least one link pointing to a type that is *a child of* the type pointed by the currently processed temporary link. When this count is one, the currently processed temporary link is removed from the currently processed class.
- Step 4: This step is executed as a per class depth first process over the class tree. Each temporary link pointing to a type that is *a child of* the type pointed by any of the links in the parent classes of the currently processed class are removed.
- Step 5: The remaining temporary links and the newly calculated *unique type links* are stored. The temporary links are cleaned from the class tree to enable the system to process the next predicate.

Results are stored in a local triple store that contains the *unique type links* and their count (number of *type links* associated to them) together with their forward and reverse multiplicities.

**Fig. 3:**
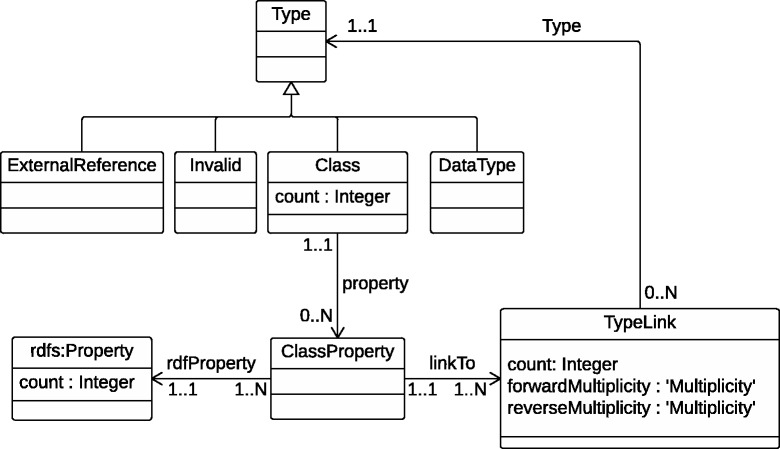
RDF2Graph ontology. A simplified UML diagram of the RDF2Graph ontology

To store information for the new concept of *unique type links* we developed a new ontology. Figure 3 depicts the elements within this ontology that are related to storage of the *unique type links*. Each *unique type link* links to an object *type* which is either: i) a class; ii) a data type, such as xsd:integer; iii) external, a subject in another resource; or iv) invalid, a subject with no defined type. In each class the *class property* groups the associated *unique type links* per predicate and links them to the rdfs:Property. Additionally, the number of occurrences are stored for each class and predicate.

## Results

**RDF2Graph** recovers the structure of an RDF triplestore endowed with a SPARQL 1.1 endpoint. The results are stored in a local triple store and can be exported to RDF, XGMML, OWL or ShEx files.

**Fig. 4:**
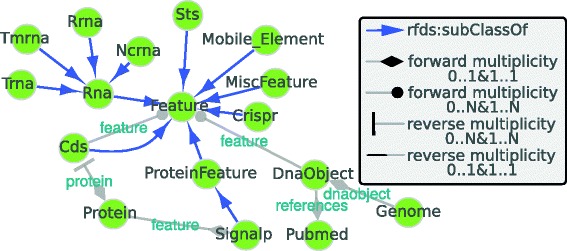
SAPP resource. Network based view generated using RDF2Graph of a resource with genome annotation generated using SAPP. Nodes represent types. Blue edges represent *subClassOf* relationships; Grey edges represent *unique type links*. Arrow heads indicate the multiplicity of the links (see legend in figure). For clarity nodes representing data types have been hidden

The RDF export contains the information on the *unique type links*, their count (number of type links associated to them) and their multiplicities (forward and reverse) as stored in the local triple store. The XGMML file provides a graphical format for a network representation that can be opened with tools such as Cytoscape. In the network each node represents a type and edges represent either *unique type links* or *subClassOf* relationships, see Fig. 4 for additional details. The associated additional information (instance count, forward/reverse multiplicity and full IRI) are stored as node and edge attributes. The XGMML exporter reports on the *unique type links* for which the multiplicity could not be determined, these correspond to *unique type links* referencing an invalid subject involved in a set of triples but without a defined type. This can be seen as a measure of the structural integrity of the resource. Additionally, the XGMML exporter reports on i) predicates joined to an invalid subject with properties but no class type definition, and ii) predicates also defined as classes, for instance using CDS (coding sequence) both as a class and a predicate

The OWL representation of the recovered structure contains the following definition and axioms: i) object and data properties, including domain and range definitions, ii) class definition, including the *all values from* restrictions to express *type links* with associated forward and, optionally, inverse cardinalities, and iii) the subclass of definitions from the original recovered resource.

The ShEx compact format contains the shape definitions with the associated class properties, *type links* and forward multiplicities. If a class property contains multiple *type links* an *or* group is included. Even though ShEx is not yet a fully mature standard, it provides better representation of the *unique type links* than other standards such as OWL. This representation is also more compact and intuitive than the generally used representations of OWL.

## Use cases

We successfully applied RDF2Graph to recover the structure of Uniprot, EBI Reactome (BioPAX level 3), ChEBI and the RDF dataset generated by SAPP, a semantic, web based, genome annotation tool currently being developed in our group. The statistics of this process are presented in Table 1.

**Table 1:** Summary statistics. Summarizing statistics of the recovery process and recovered structured for UniProt RDF, ChEBI RDF, Reactome Biopax level 3 and local resource generated by the SAPP tool

	UniProt	ChEBI	Reactome	SAPP
#unique triples in the RDF resource	16.313.400.275	425.256.854	14.285.722	359.141
CPU time needed for the recovery	742 h	6,5 h	0,5 h	2 min
#triples in local triplestore				
before simplification	2.507.483	2.854.295	2.411	1410
after simplification	2.504.259	2.848.491	964	912
#classes				
with instances	169	123	45	17
without instances	1.232.947	1.423.143	25	1
#*unique type links*				
before simplification	724	942	254	137
after simplification	302	187	69	78
multiplicity of *unique type links* after simplification				
1..1	53	77	29	46
1..n	11	1	5	2
0..n	104	27	17	4
0..1	128	78	18	26
not determined	6	4	0	0

The RDF version of the UniProt database contains more than 16 billion triples which is more than one thousand times the number of triples present in the RDF version of Reactome. Therefore there are huge differences in the time required for the recovery process. More than 99 % of the CPU time spent in the recovery process is consumed by the SPARQL endpoint.

Given the size of the UniProt resource, we had to impose a limitation on the number of considered *type links* per predicate (100.000). With this limitation the recovery process required more than 740 hours (4 days on 8 cores on a 2.3 GHz computer). On the other hand, the time required to retrieve the structure of Reactome was of only half an hour. This shows that the relationship between the recovery time and the number of triples is non linear. This nonlinearity can be attributed to the higher memory requirements associated with a larger resource but also to the intrinsic differences in the structure of the resource, given by the different values of the total number of *unique type links* and the average number of *type links* per class property (see Table 1).

Even though the UniProt RDF resource has around 40 times more triples than ChEBI RDF, they have a similar number of triples in the local resource. This is due to the high number of *concept classes* and subClassOf relationships that can be found in ChEBI, for example the subClassOf relationship associated with *galactose is an aldohexose*.

The number of triples in the local triple store does not necessarily grow with the number of triples in the resource, since the number of triples in the local triple store is associated with the complexity and the number of classes in the resource, but not with the number of occurrences of each *unique type link*. Table 1 shows that the number of triples in the local triple store is roughly equal to twice the number of classes in the resource plus eight times the number of *unique type links*. The number of classes and relationships that can be recovered is limited by the amount of triples that the local triple store can handle. In our case (Jena TDB) that would correspond to an upper limit of roughly 10^7^*unique type links* and classes. So, in practice, the only limitations are given by the restrictions on the SPARQL endpoint imposed by data providers and not by the storage capacity in the local triple store.

**Fig. 5:**
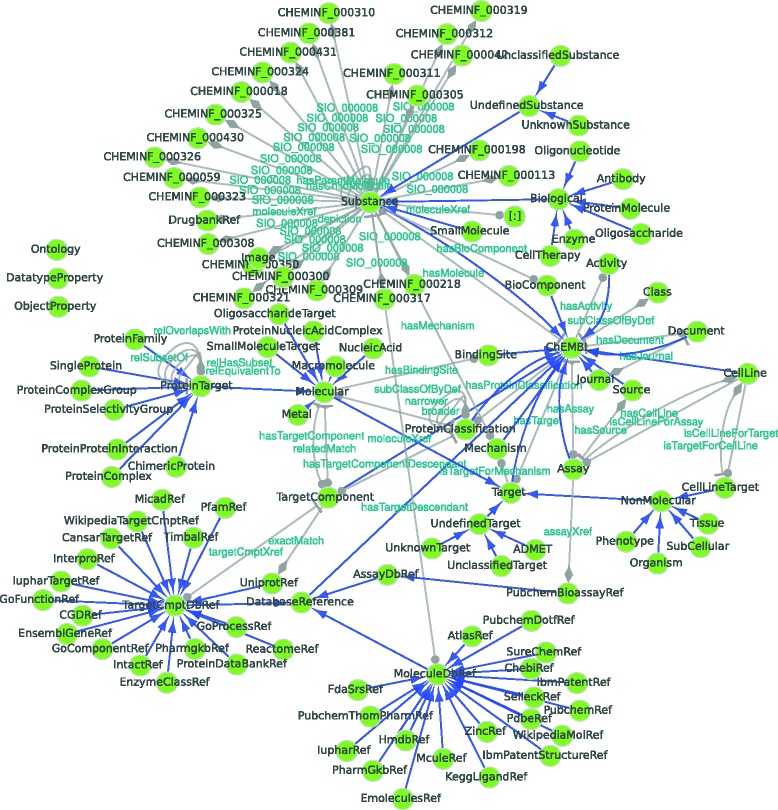
ChEBI database. Network based view generated using RDF2Graph of the ChEBI RDF resource. See Fig. 4 for legend. An interactive XGMML file is included in Additional file 1

**Fig. 6:**
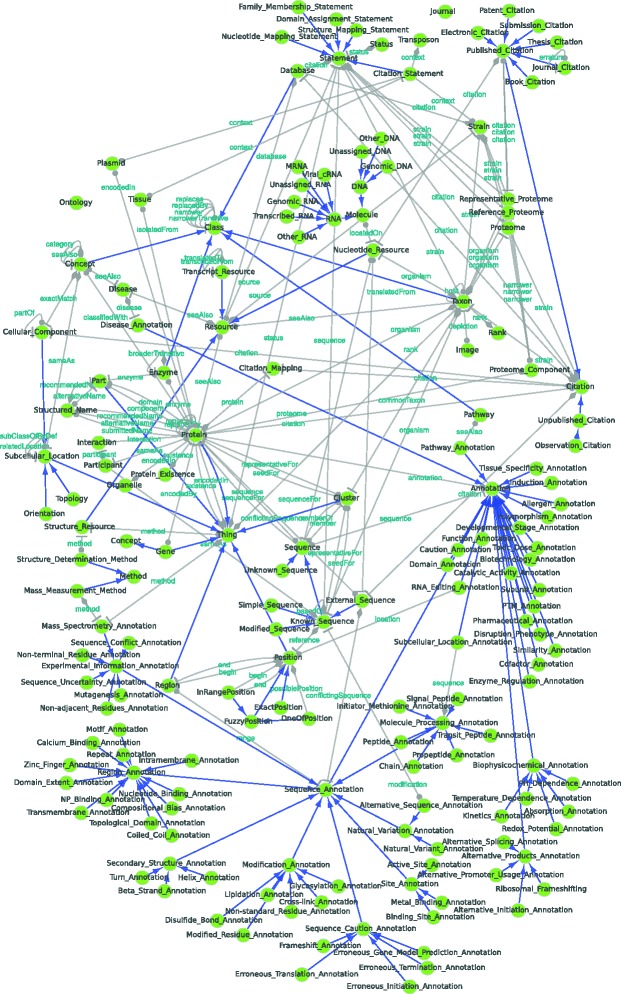
UniProt database. Network based view generated using RDF2Graph of the UniProt RDF resource. See Fig. 4 for legend. An interactive XGMML file is included in Additional file 1

**Fig. 7:**
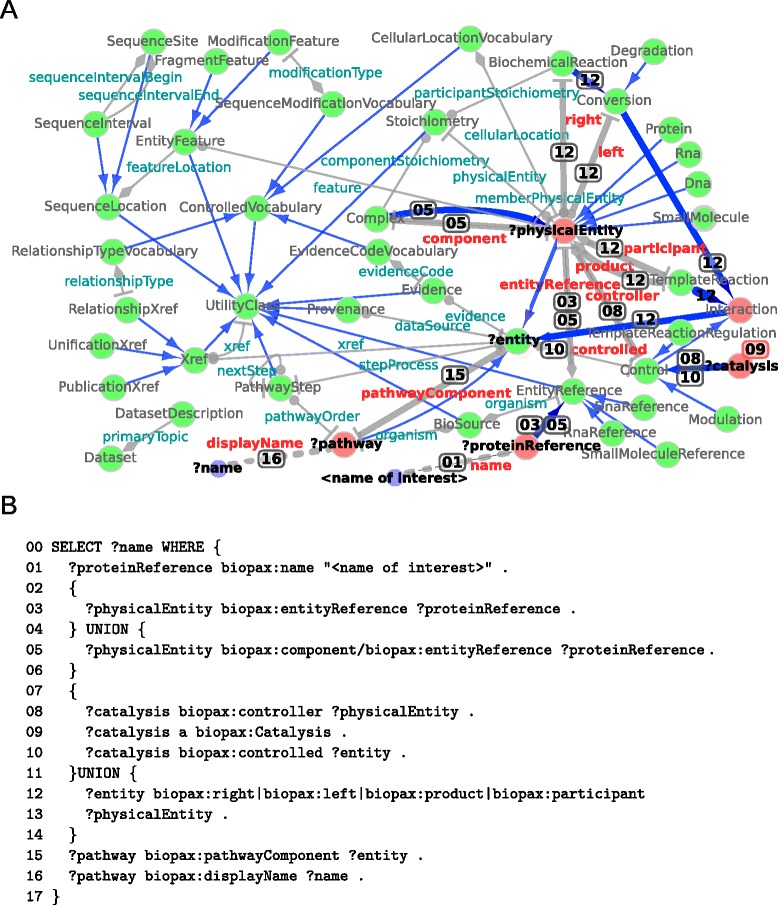
Reactome database. **a** Network based view generated using RDF2Graph of the Reactome (Biopax level 3) resource. See Fig. 4 for the legend. An interactive XGMML file is included in Additional file 1. Edges used for the query in B are highlighted. Numbers on highlighted edges correspond to line numbers in B; **b** SPARQL query to extract the names of all pathways associated to a given gene identifier

Figures 4, 5, 6 and 7 provide graphical representations of the retrieved structures for these resources (SAPP, ChEBI Uniprot and Reactome) respectively. The nodes in these representations correspond to classes with instances, whereas the edges represent the *unique type links* with determined multiplicity. See Additional file 1 for additional output of RDF2Graph regarding these resources (OWL, XGMML, ShEx and the RDF of the local store).

The simplification process reduces the number of *unique type links* from 40 % to 80 % in the selected resources. Thereby providing a neat structural overview that can be browsed for query building.

There is a small subset of *unique type links* for which the multiplicity could not be determined. These correspond to *unique type links* referencing to an invalid subject involved in a set of triples but without a defined type. These links were identified using the XGMML exporter.

We used RDF2Graph to incrementally develop and improve SAPP, our semantic annotation tool. For each incremental improvement we recovered the structure and used the graphical overview to assess the integrity of the resource and compare its intended and the actual content. We manually verified every class, associated properties and type links to identify and solve a number of issues, such as capitalization errors, predicate naming errors, faulty URI’s, broken links, missing attributes and type definitions, unwanted interconnections and faulty multiplicities. For example, a broken link will appear as a reference to an external resource where a reference to another class would be expected. A predicate naming error in one of the RDF exporter functions will cause some subjects to be in triples with the “wrong” predicate and will change the multiplicity from *1..1* to *0..1*. Finally, the OWL exporter was used to generate an OWL file requiring little manual curation to complete it.

Similarly we verified the structure of the UniProt RDF resource. The XGMML exported reported 24 issues, most of them associated to subjects with the same missing class definition (see Additional file 1). Additionally we manually compared the provided OWL file with the one created by the RDF2Graph OWL exporter. We detected a set of practical issues such as missing type definitions, references linking the wrong type of objects, incorrect multiplicities and mismatches between the descriptive OWL file and the actual content. These have been reported and will result in an improvement of the quality of this important resource.

The recovered structures and their associated statistics about classes, predicates and *type links* were successfully used to create multiple complex queries. For instance, the retrieved structure of Reactome is depicted in Fig. 7, panel A. Using this structural information we created the query in Fig. 7, panel B. This query extracts from Reactome the names of all pathways associated to a specific gene identifier. Through the use of the structural overview we were able to find and follow multiple links from the gene or protein of interest to the associated pathways.

## Discussion

RDF resources of biological data are on-going efforts, producing resources that are constantly updated and incorporating additional data sets. Detailed knowledge of the current structure is essential to query and validate these resources and RDF2Graph can be used to understand and improve the quality of an RDF resource. This becomes even more important when the goal is to perform a federated query that spans multiple RDF resources.

Our tool is complementary to existing tools that help create queries such as SPARQL assist [23], Visor [24] iSPARQL [25] and SPARQLGraph [26], these tools are based on local instance or class relationship browsing, or on query suggestion and completion or on a graphical representation of the SPARQL query.

RDF2Graph can be used to inspect instance data (also called *A box*) and semi automatically generate a descriptive OWL ontology. However, it does not check or quantify the quality of the underlying class structure and descriptions (also called *T box*). Nevertheless, there exist several tools such as OntoQA [27], OOPS [28] and OQuaRE [29]) to perform these tasks.

In the provided use cases we performed a manual structural integrity verification. If needed integrity constraint (IC) validation [30] can be used to automatically perform this task. However, for this task an OWL file with all the required axioms is needed. Such an OWL can be generated with RDF2Graph. However, the performance on the generated OWL files upon IC validation implemented by Stardog has not been extensively tested. Additionally ShEx validators, when operational, can be used for this automatic validation, however the output of RDF2Graph ShEx exporter will need to be adapted to the latest definition of ShEx.

So far, RDF2Graph does not support the recovery of contextual links (RDF quads) as they are not supported by the OWL standard although active research is being done to solve this issue [31].

## Conclusion

RDF2Graph facilitates the creation of high quality resource descriptions, which in turn improves the quality of the resources themselves. It also facilitates the creation of complex queries, therefore our tool will be helpful for improving the usability of semantic web technologies, which is required for data integration in (computational) biology, systems biology and the emerging field of semantic systems biology.

## Availability and requirements

RDF2Graph is distributed under MIT license and it is freely available from https://github.com/jessevdam/RDF2Graph. RDF2Graph is written in Java, SPARQL and bash. RDF2GRaph runs under Linux and Mac OS X (tested under version 10.11) however, a virtual machine is also distributed with the version described in this manuscript. Furthermore a Galaxy interface is available at http://semantics.systemsbiology.nl/RDF2Graph/. The RDF resource size, in this case, is limited to 20.000.000 lines.

Maven 2 [32] is required for installation and the resulting jar can be executed with Java using bash or alike. In addition it requires Jena to host the local temporary RDF store and Cytoscape (version 3.x) [21] to generate the network based overview. See the RDF2Graph manual enclosed in the Git repository for more details.

## Additional file

Additional file 1
**Output files.** Each output format of the following RDF resources: i) the CHEBI database, ii) Reactome (Biopax level 3) database, iii) UniProt database, iv) SAPP. (1,572 kb)
